# Systemic Antibiotics Influence Periodontal Parameters and Oral Microbiota, But Not Serological Markers

**DOI:** 10.3389/fcimb.2021.774665

**Published:** 2021-12-24

**Authors:** Elisa Kopra, Laura Lahdentausta, Milla Pietiäinen, Kåre Buhlin, Päivi Mäntylä, Sohvi Hörkkö, Rutger Persson, Susanna Paju, Juha Sinisalo, Aino Salminen, Pirkko J. Pussinen

**Affiliations:** ^1^ Oral and Maxillofacial Diseases, University of Helsinki and Helsinki University Hospital, Helsinki, Finland; ^2^ Division of Periodontology, Department of Dental Medicine, Karolinska Institutet, Huddinge, Sweden; ^3^ Institute of Dentistry, University of Eastern Finland, Kuopio, Finland; ^4^ Oral and Maxillofacial Diseases, Kuopio University Hospital, Kuopio, Finland; ^5^ Medical Microbiology and Immunology, Research Unit of Biomedicine, University of Oulu, Oulu, Finland; ^6^ Medical Research Center, Oulu University Hospital and University of Oulu, Oulu, Finland; ^7^ Department of Periodontics, University of Washington, Seattle, WA, United States; ^8^ Department of Oral Medicine, University of Washington, Seattle, WA, United States; ^9^ Faculty of Health Sciences, Kristianstad University, Kristianstad, Sweden; ^10^ Division of Cardiology, Heart and Lung Center, Department of Medicine, Helsinki University Hospital, Helsinki, Finland

**Keywords:** periodontitis, antibiotics, oral microbiota, oral microbiome, immune response, antibodies, saliva, periodontal pathogens

## Abstract

The use of systemic antibiotics may influence the oral microbiota composition. Our aim was to investigate in this retrospective study whether the use of prescribed antibiotics associate with periodontal status, oral microbiota, and antibodies against the periodontal pathogens. The Social Insurance Institution of Finland Data provided the data on the use of systemic antibiotics by record linkage to purchased medications and entitled reimbursements up to 1 year before the oral examination and sampling. Six different classes of antibiotics were considered. The Parogene cohort included 505 subjects undergoing coronary angiography with the mean (SD) age of 63.4 (9.2) years and 65% of males. Subgingival plaque samples were analysed using the checkerboard DNA-DNA hybridisation. Serum and saliva antibody levels to periodontal pathogens were analysed with immunoassays and lipopolysaccharide (LPS) activity with the LAL assay. Systemic antibiotics were prescribed for 261 (51.7%) patients during the preceding year. The mean number of prescriptions among them was 2.13 (range 1–12), and 29.4% of the prescriptions were cephalosporins, 25.7% penicillins, 14.3% quinolones, 12.7% macrolides or lincomycin, 12.0% tetracycline, and 5.8% trimethoprim or sulphonamides. In linear regression models adjusted for age, sex, current smoking, and diabetes, number of antibiotic courses associated significantly with low periodontal inflammation burden index (PIBI, *p* < 0.001), bleeding on probing (BOP, *p* = 0.006), and alveolar bone loss (ABL, *p* = 0.042). Cephalosporins associated with all the parameters. The phyla mainly affected by the antibiotics were Bacteroidetes and Spirochaetes. Their levels were inversely associated with the number of prescriptions (*p* = 0.010 and *p* < 0.001) and directly associated with the time since the last prescription (*p* = 0.019 and *p* < 0.001). Significant inverse associations were observed between the number of prescriptions and saliva concentrations of *Prevotella intermedia*, *Tannerella forsythia*, and *Treponema denticola* and subgingival bacterial amounts of *Porphyromonas gingivalis*, *P. intermedia*, *T. forsythia*, and *T. denticola*. Saliva or serum antibody levels did not present an association with the use of antibiotics. Both serum (*p* = 0.031) and saliva (*p* = 0.032) LPS activity was lower in patients having any antibiotic course less than 1 month before sampling. Systemic antibiotics have effects on periodontal inflammation and oral microbiota composition, whereas the effects on host immune responses against the periodontal biomarker species seem unchanged.

## Introduction

Periodontitis is among the most common bacterial infection in humans. It is an inflammatory disease of the supporting tissues of the teeth, initiated by microorganisms in the dental plaque, resulting in progressive destruction of the connective tissue and bone support. The host response to bacterial insult leads to gingival swelling, bleeding on gentle probing, increased periodontal pocket depth, and alveolar bone loss. Finally, untreated periodontitis may lead to the loss of teeth.

To control the periodontal disease, it is mandatory to recognise the pathogenic role of bacteria that accumulate in the periodontal pocket. Usually, during the progression of bacterial infections, the diversity of the microbiota decreases. However, in periodontitis, the diversity of the flora increases (Mombelli, 2018), and subgingival plaque harbours the highest richness and diversity of species in the oral cavity. The phyla comprising 99% of the total counts are typically Gram-negative Bacteroidetes, Fusobacteria, Proteobacteria, Saccharibacteria, and Spirochaetes and Gram-positive Firmicutes and Actinobacteria (Chen et al., 2018). The Gram-negative anaerobic bacteria, the keystone pathogens for periodontitis, promote inflammation and dysbiosis in susceptible individuals (Hajishengallis and Chavakis, 2021). The mechanical removal of calculus and biofilm (scaling and root planing) from the tooth surface is the primary method for treating periodontitis. The use of systemic antibiotics may suppress specific microorganisms within the oral cavity and especially in periodontal pockets. However, there is no data that selective suppression of any single member of periodontal pocket microbiota would be the key to success of periodontal therapy, because dysbiosis is the problem (Mombelli, 2018).

The effects of systemic antibiotics to the composition of oral microbiota are not very well described. Even though the oral cavity is the starting point of gastrointestinal tract, most of the studies are concentrated on the gut microbiota (Yang et al., 2021). In the gut, antibiotics disrupt both short- and long-term microbial conditions and diversity (Clemente et al., 2012), but the length of time to recovery can vary individually (Yang et al., 2021). The previous research has focused mostly on usage of adjunctive systemic antibiotics in the periodontal treatment. However, no data are available on the effect of using systemic antibiotics prescribed for other clinical indications than periodontitis on the composition of oral microbiota. The aim of the study was to investigate whether the use of prescribed antibiotics associate with periodontal status, oral microbiota, and antibodies against the periodontal pathogens. This retrospective study covered antibiotic purchases up to 1 year before the oral examination and microbial sampling.

## Methods

### Population

The Finnish Corogene is a prospective cohort designed to study coronary artery disease (CAD) and other related heart diseases. It includes 5,297 consecutive patients undergoing coronary angiography at the Helsinki University Hospital between June 2006 and March 2008 (Vaara et al., 2012). The Parogene cohort is a random gender-stratified subpopulation of the Corogene and consists of 508 patients enrolled for extensive clinical and radiographic oral examination 6–20 weeks after the angiography. The detailed description of the data collection including the oral examinations has been described earlier (Buhlin et al., 2011). From the original cohort of 508 participants, 3 (0.6%) were lost to follow-up leading to a final number of 505 participants. The study complies with the principles of the Declaration of Helsinki, and written informed consent was obtained from all study subjects. The Parogene study design was approved by the Helsinki University Hospital ethics committee (Reg. no. 106/2007).

### Oral Examination

The patients filled a questionnaire including details of their smoking habits and visits to a dentist. Two calibrated periodontal specialists performed the oral examination recording bleeding on probing (BOP) and probing pocket depth (PPD) from six sites of all teeth. BOP is presented as percentage of bleeding sites from all sites examined, and PPD was categorised to number of probing depth exceeding 4 or 6 mm. The periodontal inflammatory burden (PIBI) was calculated as (PPD ≥4 mm + 2 × PPD ≥6 mm) (Lindy et al., 2008).

Panoramic radiographs were used to register data on number of teeth with caries and apical rarefactions (Liljestrand et al., 2016; Liljestrand et al., 2021) and alveolar bone loss (ABL) (Buhlin et al., 2011).

The information on the use of antibiotics is based on data linkage to nationwide registers using the individual social security number. The data of medication usage comes from the Finnish prescription register maintained by the Social Insurance Institution of Finland. This registry covers all patients’ medication purchases from the 1st of January 2005, which is >1 year before the initial hospitalisation (Vaara et al., 2012). The antibiotic information was collected from registry by using Anatomical Therapeutic Chemical (ATC) classification code and data of systemic antimicrobial class J were collected. Patients were found to have used the following antibiotic groups: tetracyclins, penicillins, cephalosporins, trimethoprim, macrolides/lincomycin, and quinolones. The ATC group, action mechanism, and spectrum of these antibiotics are listed in **Supplementary Table S1**.

### Characterisation of Subgingival Microbiota

The details of the subgingival bacterial sampling and characterisation have been published earlier (Mäntylä et al., 2013; Pradhan-Palikhe et al., 2013). Briefly, the deepest pathological pocket (PPD≥ 4 mm) in each dentate quadrant was chosen for bacterial sampling. The sample was taken with a sterile curette after isolation of the site from saliva and removing supragingival plaque. The deepest possible sites with signs of inflammation (with BOP) were selected for sampling in patients with no sulci exceeding a depth of 3 mm. The samples were pooled within a patient and frozen. The microbiological analyses were performed using the DNA-DNA hybridisation assay (Socransky et al., 2004). The results were obtained after comparing the signals to standard lanes and presented as counts of bacteria × 10^5^. The results of single species were summed up within the phylum. The list of the species, phyla, and related information is presented as **Supplementary Table S2**.

### Determination of Concentrations of Periodontal Bacteria

The bacterial strains used as antigens were following: *A. actinomycetemcomitans*, ATCC29523, ATCC43718, ATCC3384, IDH781, IDH1705, C59A; *P. gingivalis*, ATCC33277, W50, OMGS434; *P. endodontalis*, ATCC35406; *P. intermedia*, ATCC25611; and *T. forsythia*, ATCC43037.

Stimulated saliva samples were collected by expectoration before the oral examination. The collection time was 5 min or the time to obtain at least 2 ml of saliva. The samples were frozen at −70°C and DNA was isolated from pellets after 3-min centrifugation at 9,300×*g*. The saliva collection and processing has been described earlier in detail (Hyvärinen et al., 2012). Concentrations of *A. actinomycetemcomitans*, *P. gingivalis*, *P. intermedia*, *T. forsythia*, and *T. denticola* were determined by quantitative real-time PCR assays as described in detail in our earlier publications (Hyvärinen et al., 2009; Pietiäinen et al., 2018). After comparing to the standard reference strains, the results were presented as genomes/ml (GE/ml).

### Determination of Saliva and Serum Antibody Levels Against Periodontal Species

The serum levels of IgA and IgG antibodies against the whole-cell antigens of *A. actinomycetemcomitans*, *P. gingivalis*, *P. endodontalis*, *P. intermedia*, and *T. forsythia* were determined as described in detail earlier (Liljestrand et al., 2018) using multiserotype ELISA (Pussinen et al., 2002; Pussinen et al., 2011).

The saliva levels of IgA and IgG antibodies against the whole-cell antigens of *A. actinomycetemcomitans*, *P. gingivalis*, *P. endodontalis*, *P. intermedia*, and *T. forsythia* were determined as described in detail earlier using chemiluminescence assays (Akhi et al., 2019).

To show how the serum and saliva antibody levels to Bacteroidetes species relate to the use of antibiotics, the antibody levels of *P. gingivalis*, *P. endodontalis*, *P. intermedia*, and *T. forsythia* were summed up.

### Statistical Analyses

The variables with skewed distribution, such as the antibody and bacterial levels, were log-transformed before statistical analyses. For the tables, the values were back-transformed. We used 10-based logarithm for the bacterial levels and natural logarithm for the antibody levels. The statistical significance between the groups was tested using either *t*-test or ANOVA. The levels are presented as mean with standard error of mean (SE). The *post-hoc* test was LSD. The significance of categorical parameters was tested using Chi-square. Linear regression models adjusted for age and sex were used to examine the associations between parameters. The models concerning periodontal parameters were additionally adjusted for smoking (never/ever) and diabetes (no/yes).

## Results

**Figure 1:**
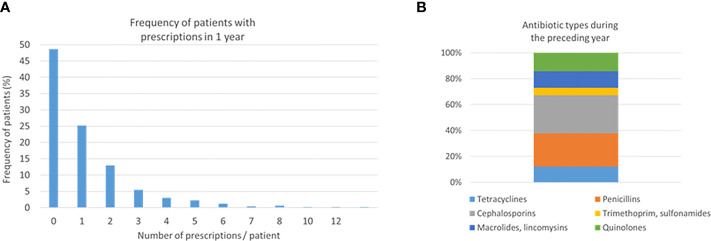
Number of prescriptions and antibiotic types. Two hundred sixty-one (51.7%) patients were prescribed with antibiotics during the preceding 1 year. Frequencies of the number of prescriptions **(A)** and antibiotic types **(B)** are shown.

From the available cohort of 505 participants, 261 (51.7%) patients were prescribed with antibiotics during the preceding 1 year. The mean number of prescriptions among them was 2.13 (range 1–12) (**Figure 1A**). A total of 29.4% of the prescriptions were cephalosporins, 25.7% penicillins, 14.3% quinolones, 12.7% macrolides or lincomycin, 12.0% tetracycline, and 5.8% trimethoprim or sulphonamides (**Figure 1B**). Women had more often trimethoprim or sulphonamides than men (9.1% vs. 2.5%, *p* = 0.042) (**Supplementary Figure S1**). The main characteristics of these antibiotic classes are listed in **Supplementary Table S1**. Cephalosporins are mainly used to treat staphylococcal, streptococcal, and pneumococcal infections especially in the case of penicillin allergy. Quinolones are mainly prescribed not only to treat severe bacterial infections but also to treat and prevent intestinal and urinary tract infections. Other antibiotic classes have less indications for use such as *Helicobacter* eradication (tetracyclins), urinary tract infections (trimethoprim), and respiratory infections caused mainly by *Mycoplasma pneumoniane* or *Chlamydia pneumoniae* (macrolides/lincomycin).

**Figure 2:**
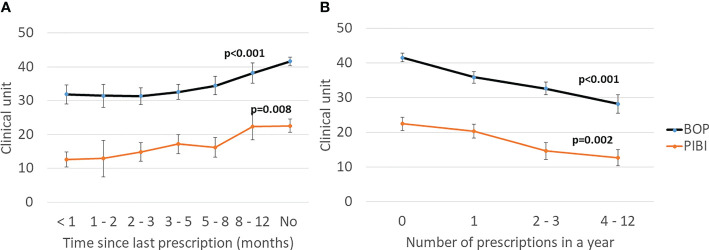
Effect of antibiotics on clinical periodontal parameters. **(A)** Time since the last prescription. **(B)** Number of prescriptions during the preceding year. Mean values with the SE are shown. Bleeding on probing (BOP) is the percentage of bleeding sites from all examined sites. Periodontal inflammatory burden index (PIBI) is the number of deepened periodontal pockets: PPD ≥4 mm + 2 × PPD ≥6 mm. The p-values are for the weighted linear terms from Anova for log-transformed values.

The characteristics of the study participants stratified according to the use of antibiotics are presented in **Table 1**. The antibiotic users had higher BMI and more often diabetes than the nonusers. Regarding their oral parameters, the antibiotic users had lower BOP, fewer sites with PPD ≥6 mm, lower PIBI, and less-severe ABL. The data stratified by sex is presented in the supplement (**Supplementary Table S3**). The levels of BOP and PIBI are shown according to the time since last prescription and number of prescriptions during the year in **Figure 2**. After adjusting for relevant confounders, the number of antibiotic prescriptions for any clinical purpose during the preceding year was significantly and inversely associated with BOP, PIBI, and ABL (**Table 2**). BOP, PIBI, and ABL were inversely associated with the number of prescribed cephalosporin courses. In addition, PIBI was associated with the number of quinolone prescriptions and BOP with penicillin and macrolides/lincosamide.

**Table 1:** Characteristics of the population stratified by the use of antibiotics during the preceding year.

Parameter		No antibiotics (*n* = 244)	Antibiotics (*n* = 261)
		Mean (SD)		*p*-value^a^
Age (years)		63.8 (9.3)	62.9 (9.0)	0.242
BMI (kg/m^2^)		27.4 (4.6)	28.3 (5.4)	**0.042**
Number of teeth	x-ray	19.6 (9.2)	20.3 (8.6)	0.353
Caries (*n* of teeth)^b^	x-ray	1.02 (1.69)	0.92 (1.29)	0.956
Apical rarefactions (n of teeth)^b^	x-ray	0.46 (1.19)	0.27 (0.58)	0.427
BOP (% of sites)^b^	Clinical	41.6 (19.5)	33.6 (17.6)	**<0.001**
PPD ≥4 mm (n of sites)^b^	Clinical	14.0 (14.0)	12.2 (12.2)	0.346
PPD ≥6 mm (n of sites)^b^	Clinical	4.26 (9.84)	2.47 (6.38)	**0.042**
PIBI^b^	Clinical	22.5 (29.5)	17.1 (21.5)	**0.049**
		***N* (%)**		*p*-value^c^
Sex (females)		81 (33.2)	96 (36.8)	0.399
Smoking (ever)^d^		118 (51.8)	132 (54.3)	0.577
Periodontal treatment^d^		18 (8.1)	37 (15.9)	**0.011**
Diabetes		48 (19.7)	70 (27.2)	**0.046**
Alveolar bone loss	No	48 (21.1)	65 (26.4)	**0.018**
	Mild	97 (42.5)	116 (47.2)	
	Moderate	63 (27.6)	58 (23.6)	
	Severe	20 (8.8)	7 (2.8)	
Edentulous		17 (7.0)	15 (5.7)	0.574

a t-test.

b Log-transformation, mean, and SD after back-transformation.

^c^Chi-square.

^d^Based on questionnaire on smoking and response to a question, whether the patient has ever received any periodontal treatment.

Significant p-values are in bold face.

**Table 2:** Association of antibiotic use with periodontal parameters.

Number of antibiotics prescriptions in a year	BOP		PIBI		ABL	
	*B* (SE)	*p*-value	*B* (SE)	*p*-value	*B* (SE)	*p*-value
Any	−2.82 (0.57)	**<0.001**	−2.02 (0.73)	**0.006**	−0.05 (0.022)	**0.042**
Tetracycline	−1.45 (2.38)	0.542	0.39 (2.61)	0.880	−0.04 (0.08)	0.638
Penicillin	−4.87 (1.47)	**0.001**	−1.22 (1.73)	0.482	−0.08 (0.05)	0.137
Cephalosporin	−3.78 (1.23)	**0.002**	−3.84 (1.62)	**0.018**	−0.13 (0.05)	**0.007**
Trimethoprim/sulfa	−4.36 (2.89)	0.131	−2.61 (3.84)	0.498	−0.02 (0.12)	0.856
Macrolides/lincosamide	−4.95 (1.59)	**0.002**	−3.60 (2.11)	0.089	−0.02 (0.07)	0.718
Quinolones	−3.26 (1.84)	0.077	−5.43 (2.38)	**0.023**	0.03 (0.07)	0.700

Linear regression models adjusted for age, sex, smoking (never/ever), and diabetes (no/yes).

BOP, bleeding on probing, % of bleeding surfaces; PIBI, periodontal inflammatory burden index, [PPD ≥4 mm + 2 × (PPD ≥6 mm)]; ABL, alveolar bone loss from none to severe (0–4).

Significant p-values are in bold face.

**Figure 3:**
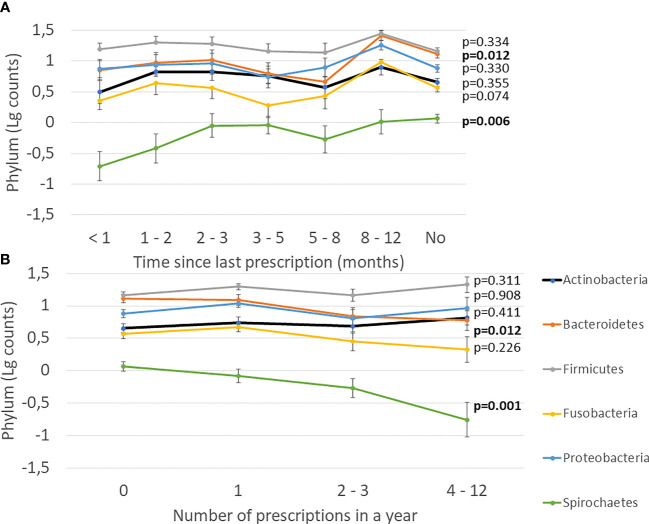
Effect of antibiotics on subgingival microbiota. **(A)** Time since the last prescription. **(B)** Number of prescriptions during the preceding year. Mean values of log-transformed bacterial counts on the phylum level with the SE are shown. The p-values are for the weighted linear terms from Anova.

**Figure 4:**
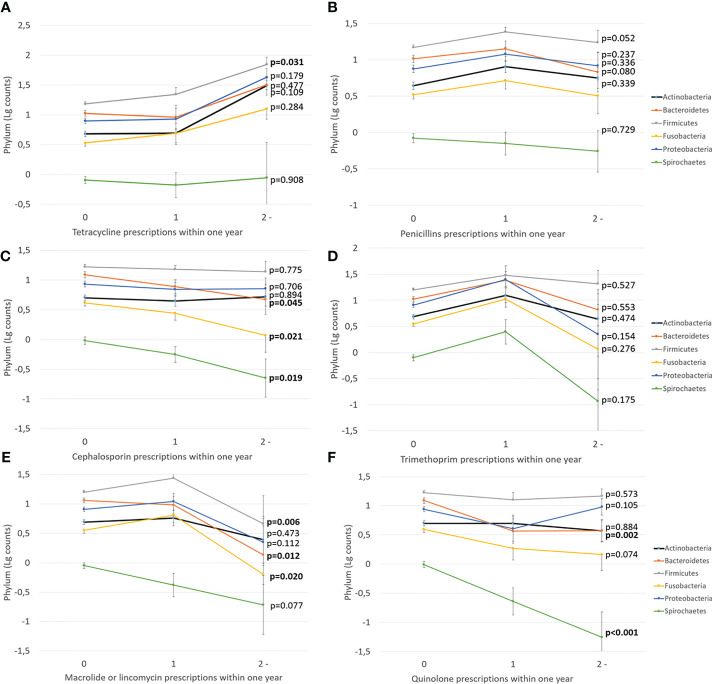
The effect of different antibiotic types on subgingival microbiota. Mean values of log-transformed bacterial counts on the phylum level with the SE are shown. The p-values are for the weighted linear terms from Anova. The antibiotic classes are: **(A)** tetracycline, **(B)** penicillins, **(C)** cephalosporin, **(D)** trimethoprim, **(E)** macrolide/lincomycin, and **(F)** quinolone.

Next, we investigated the effect of antibiotics on subgingival microbiota. The time since the last prescription and the number of prescriptions affected significantly the levels of Bacteroidetes and Spirochaetes, whereas other phyla, Actinobacteria, Firmicutes, Fusobacteria, and Proteobacteria, remained unaffected (**Figure 3**). Furthermore, we investigated the effect of types and amounts of different antibiotics on the subgingival microbiota (**Figure 4**). The amount of Bacteroidetes was lower among users of macrolide/lincomycin or quinolone; Fusobacteria was lower among users of cephalosporin or macrolide/lincomycin; Spirochaetes was lower among users of cephalosporin or quinolone; and Firmicutes was lower in users of macrolide/lincomycin and higher among users of tetracycline. None of the antibiotics affected the amount of Actinobacteria and Proteobacteria. The associations were analysed using linear regression models adjusted for age and sex (**Table 3**). The amount of Bacteroidetes was inversely associated with the use of macrolide/lincomycin and quinolone, Fusobacteria with cephalosporin, and Spirochaetes with quinolone. In addition, the positive association between Firmicutes and tetracycline was significant.

**Table 3:** Association of antibiotic type with subgingival phyla.

	*Actinobacteria*	*Bacteroidetes*	*Firmicutes*	*Fusobacteria*	*Proteobacteria*	*Spirochaetes*
	Standardised coefficient (beta) and p-value					
Time since last prescription^a^	−0.003, 0.954	**0.108, 0.019**	−0.021, 0.653	0.059, 0.199	0.010, 0.833	**0.176, <0.001**
Any antibiotics^a^	0.039, 0.403	**−0.119, 0.010**	0.043, 0.350	−0.057, 0.211	0.003, 0.948	**−0.167, <0.001**
Tetracyclines^b^	0.061, 0.190	0.027, 0.559	**0.115, 0.013**	0.080, 0.082	0.062, 0.180	0.002, 0.971
Penicillins^b^	0.082, 0.083	0.027, 0.557	0.083, 0.075	0.047, 0.309	0.059, 0.212	−0.012, 0.799
Cephalosporins^b^	−0.008, 0.874	−0.075, 0.113	−0.035, 0.467	**−0.108, 0.023**	−0.024, 0.624	−0.065, 0.163
Trimethoprim, sulphonamides^b^	0.032. 0.487	0.020, 0.660	0.042, 0.362	0.001, 0.975	−0.010, 0.833	−0.009, 0.842
Macrolides, lincomycin^b^	−0.053, 0.268	**−0.104, 0.029**	−0.057, 0.231	−0.038, 0.424	−0.059, 0.219	−0.068, 0.146
Quinolones^b^	−0.016, 0.736	**−0.123, 0.009**	−0.029, 0.538	−0.075, 0.113	−0.044, 0.353	**−0.190, <0.001**

Linear regression model adjusted for age and sex.

a Time as categories: <1 month, 1–2 months, 2–3 months, 3–5 months, 5–8 months, 8–12 months, none.

b Antibiotics are categorised as: not within 1 year; once within 1 year; ≥twice within 1 year. All bacterial levels are log-transformed.

Significant p-values are in bold face.

**Figure 5:**
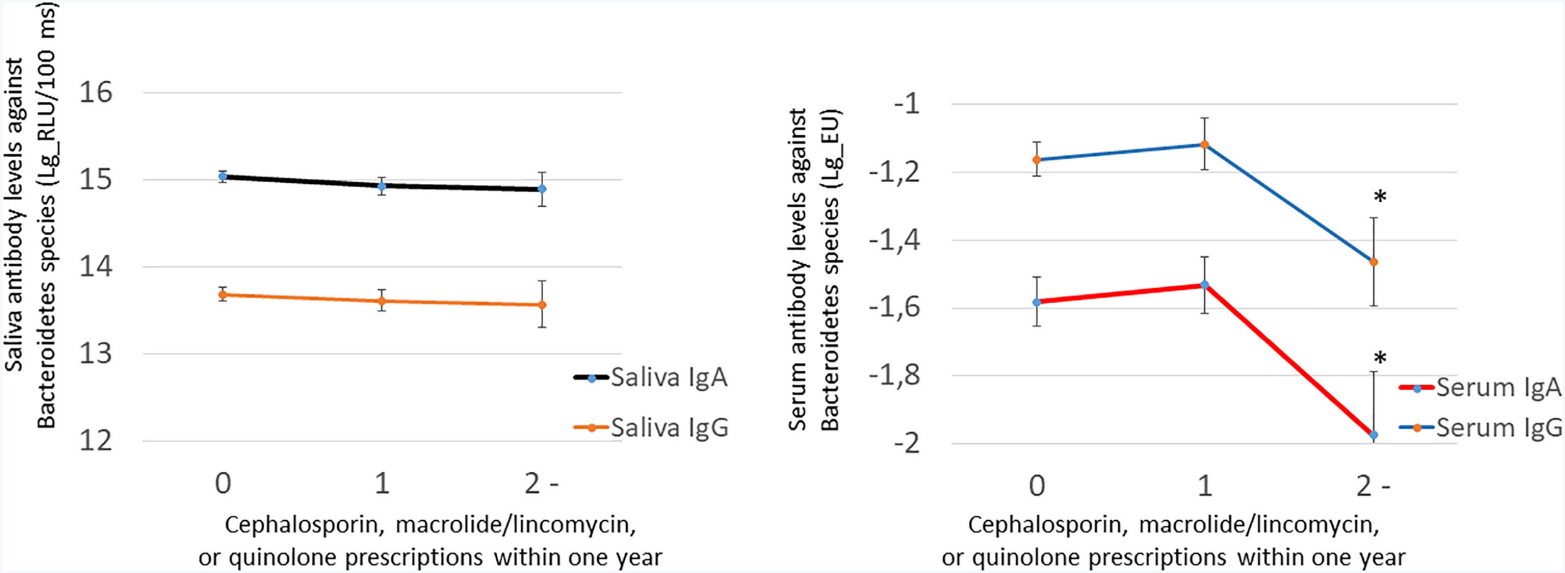
The effect of cephalosporin, macrolide/lincomycin, or quinolone use on saliva and serum antibodies against the Bacteroidetes species. Saliva antibodies were determined using a chemiluminescence immunoassay and serum antibodies using the multiserotype ELISA. Both IgA and IgG class antibodies were measured. The antibody levels against the Bacteroidetes species comprise summed values from separate assays of *P. gingivalis*, *P. endodontalis*, *P. intermedia*, and *T. forsythia*. The number of patients in the groups are 0, 286; 1, 127; and ≥2, 38. The star depicts a *p*-value <0.05 compared with nonusers of antibiotics as produced by LSD test after ANOVA.

We investigated the associations of the use of antibiotics with the levels of periodontal pathogens by linear regression models (**Table 4**). Significant inverse associations were observed between the number of prescriptions and saliva concentrations of *Prevotella intermedia*, *Tannerella forsythia*, and *Treponema denticola*. The number of prescriptions was also associated with subgingival bacterial amounts of *Porphyromonas gingivalis*, *P. intermedia*, *T. forsythia*, and *T. denticola*. While the use of antibiotics affected the subgingival and saliva bacterial levels, they did not affect serum or saliva antibody levels against these species (**Supplementary Table S4**). **Figure 4** shows the sums of antibody levels against Bacteroidetes species in the groups of patients that had used either cephalosporin, macrolides/lincomycin, or quinolones during the preceding year. Although these antibiotic types affected the Bacteroidetes phylum, they had only minor effects on the saliva or serum antibody levels against some member species of the phylum (**Figure 5**).

**Table 4:** Association of saliva and subgingival concentrations of periodontal bacteria and number of prescribed antibiotics within a year.

Periodontal pathogen in saliva (Lg GE/ml)	Saliva (Lg GE/ml)		Subgingival (Lg counts × 10^5^)	
	*B* (SE)	*p*-value	*B* (SE)	*p*-value
*Aggregatibacter actinomycetemcomitans*	−0.097 (0.060)	0.108	0.019 (0.045)	0.670
*Porphyromonas gingivalis*	−0.136 (0.117)	0.246	−0.139 (0.051)	**0.007**
*Prevotella intermedia*	−0.418 (0.104)	**<0.001**	−0.101 (0.050)	**0.045**
*Tannerella forsythia*	−0.475 (0.133)	**<0.001**	−0.304 (0.062)	**<0.001**
*Treponema denticola*	−0.327 (0.112)	**0.004**	−0.193 (0.047)	**<0.001**

Adjusted for age and sex. The use of antibiotics is categorised as: no, once, 2–3 times, and ≥4 times within a year.

Significant p-values are in bold face.

**Figure 6:**
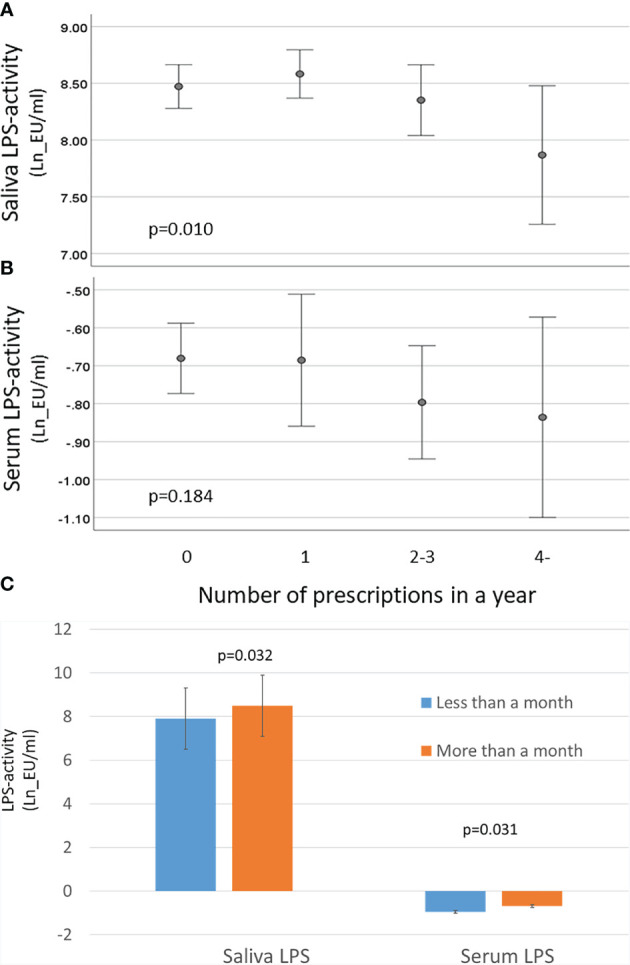
Effect of antibiotics on LPS activity. Saliva and serum LPS activity was determined by LAL assay, and the results were calculated in groups of patients having 0, 1, 2–3, or ≥4 prescriptions during the preceding year. **(A)** Mean saliva and **(B)** serum LPS activity and 95% CI are shown for logarithmically transformed values (Ln). The p-values are for the weighted linear terms from Anova. **(C)** Mean saliva and serum LPS activity (and SD) in patients having antibiotics within a month vs. more than a month ago with *p*-values from *t*-test.

The composition of subgingival microbiota contributed to saliva LPS-activity, whereas the correlation was not that obvious in serum LPS levels (**Supplementary Table S5**). Thus, we investigated whether the use of antibiotics has an effect of the LPS levels (**Figure 6**). The patients with several prescribed antibiotics during the preceding year had lower saliva LPS activity levels, but the observed trend in serum LPS levels was nonsignificant. However, the effect of antibiotics on both saliva and serum LPS level was short term, since we observed differences only up to 1 month (**Figure 6C**). The associations were analysed by linear regression models (**Table 5**). Only the inverse association between saliva LPS activity and use of quinolones was statistically significant, and the use of these antibiotics seemed to have a remarked positive association with the ratio of Gram-positive/Gram-negative bacteria and Firmicutes/Bacteroidetes (**Table 5**). Their levels are presented in **Supplementary Figure S2**.

**Table 5:** Association of antibiotic type with subgingival phyla.

	Saliva LPS (EU/ml)	Serum LPS (EU/ml)	Gram-positives (Lg_counts)	Gram-negatives (Lg_counts)	Gram-positives/Gram-negatives (Lg_counts)	*Firmicutes*/*Bacteroidetes* (Lg_counts)
	Standardised coefficient (beta) and p-value					
Tetracyclines	−0.050, 0.274	−0.022, 0.635	**0.128, 0.006**	**0.093, 0.044**	0.059, 0.194	**0.099, 0.029**
Penicillins	0.007, 0.872	0.014, 0.768	0.053, 0.254	0.048, 0.299	0.052, 0.259	0.056, 0.218
Cephalosporins	−0.036, 0.437	−0.059, 0.215	−0.058, 0.225	−0.059, 0.214	0.076, 0.108	0.042, 0.367
Trimethoprim, sulphonamides	0.005, 0.918	−0.038, 0.405	0.032, 0.482	0.005, 0.920	0.046, 0.311	0.021, 0.639
Macrolides, lincomycins	0.002, 0.959	0.006, 0.898	0.017, 0.730	−0.052, 0.279	0.030, 0.518	**0.101, 0.032**
Quinolones	**−0.126, 0.007**	−0.052, 0.264	−0.047, 0.317	**−0.113, 0.017**	**0.153, 0.001**	**0.187, <0.001**

Linear regression model adjusted for age and sex. Antibiotics are categorised as: not within 1 year; once within 1 year; ≥twice within 1 year. All bacterial levels are log-transformed. EU, endotoxin units.

Significant p-values are in bold face.

A total of 55 (10.9%) patients indicated in the questionnaire that they had received any kind of periodontal treatment (**Table 1**). These patients did not differ from the rest of the population as regards to clinical periodontal parameters, subgingival Firmicutes/Bacteroidetes ratio, or serum or saliva LPS levels (data not shown).

## Discussion

In this large, retrospective analysis, we show how the use of any systemic antibiotics prescribed for other clinical indications than periodontitis was associated with decreased levels of subgingival Bacteroidetes and Spirochaetes levels, whereas the levels of these phyla increased with the time passed since the last prescription. Although this association was obvious until 5–8 months before the sampling, we failed to see any association with the systemic or local antibody levels against the species in Bacteroidetes phyla. The use of any systemic antibiotics associated also with fewer clinical and radiographic signs of periodontitis.

The standard treatment of periodontitis is scaling and root planing (SRP), which aims at mechanically reducing the amount of biofilm and disrupting the local ecologic niche of bacteria at both supra- and subgingival areas. Despite its microbiologically unspecific nature, SRP is associated with a beneficial change in the composition of the subgingival biofilm. A variety of systemic antimicrobials have been used as adjuncts to SRP in the treatment of periodontitis, with the purpose to reach deep pockets and pathogens that may invade host tissues. A recent systematic review and meta-analysis with 24 RCTs showed that the adjunctive use of systemic antimicrobials in the active phase of periodontal treatment led to a statistically significant additional full-mouth PPD reduction (weighted mean difference = 0.448 mm) and CAL gain (0.389 mm) at 6 months, when compared with the control groups (Teughels et al., 2020). These beneficial effects of systemic antimicrobials remained stable for at least 1 year. Additionally, statistically significant benefits were also found for CAL and BOP. The best outcomes were observed for the combination of amoxicillin plus metronidazole, followed by metronidazole alone and azithromycin. Even though several meta-analyses have suggested that systemic antimicrobials may have a statistically significant effect on periodontal parameters, there has been debate about the clinical significance since the observed benefits have been relatively small when measuring mean full-mouth PPDs or attachment gain. The S3 level clinical practice guideline of the European Federation of Periodontology does not recommend the routine use of systemic antibiotics as adjunct to SRP in patients with periodontitis due to concerns to patient’s health and the impact of systemic antibiotic use to public health (Herrera et al., 2008; Sanz et al., 2020). Yet, for specific patient groups, such as young patients with severe periodontitis, the use of systemic antimicrobials may be considered. The patients of the present cohort can be regarded as “untreated for periodontitis”, although according to the questionnaire, 10.9% had received periodontal treatment sometime during their adult life. However, since their periodontal status or subgingival bacterial levels did not differ significantly from the rest of the patients and since the exact time of the received treatment was not known, we did not consider this further in the analyses. Thus, our results indicate that systemic antibiotics affect the periodontal parameters and the composition of subgingival microbiota even without mechanical instrumentation.

Most bacteria occupying periodontal pocket are living as a part of the subgingival biofilm. Bacterial cells embedded in a complex, self-produced polymeric matrix differ remarkably from planktonic cells and are more resistant to environmental changes including antibiotic treatments (Mah and O'Toole, 2001; Burmolle et al., 2014). According to a recent meta-analysis, shifts in the composition of microbial community during periodontal therapy occur regardless of the use of antibiotics as a part of the treatment. Instead of whole biofilm, systemic antibiotics seem to cause changes in the individual bacterial species (Dilber et al., 2020). Studies with *in vitro* subgingival biofilm models showed that antibiotics caused reductions in *Streptococcus anginosus*, *P. gingivalis*, and *Fusobacterium nucleatum* species, and the effect was significant by doxycycline, azithromycin, and amoxicillin alone or in combination with metronidazole, but metronidazole alone had no effect on biofilm composition (Belibasakis and Thurnheer, 2014). On the other hand, subinhibitory concentrations of antibiotics have also been reported to increase the biofilm formation in some *in vitro* biofilm models (Jin et al., 2020; Bernardi et al., 2021).

Ferrer with coworkers have listed 42 major microbial genera whose abundance is altered after treatment with any of the commonly used antibiotics. This suggests that regardless of the original disease for which they were prescribed, antibiotics can cause perturbations in bacterial communities without changing overall composition and diversity but rather affecting a specific set of bacteria. The main phyla affected are Actinobacteria, Bacteroidetes, Firmicutes, and Proteobacteria, with the most common genera influenced being *Prevotella* (Bacteroidetes), *Clostridium*, *Enterococcus*, *Lactobacillus*, *Ruminococcus*, *Streptococcus*, *Eubacterium*, (Firmicutes), and *Enterobacter* (Proteobacteria) (Ferrer et al., 2017). In the present study, significant changes in the bacterial levels associating with antibiotic usage for any purpose were found among phyla Bacteroidetes and Spirochaetes. The effect was dependent both on the number of antibiotic prescriptions and time since the last prescription. When all the six antibiotic groups were individually studied, most of the changes in the bacterial levels were associated with the number of prescriptions belonging either to cephalosporins, macrolides/lincomycin, or quinolones whereas penicillin and trimethoprim had no impact on any of the phyla. The studied species belonging to the phyla Bacteroidetes and Spirochaetes included several bacterial species associating with the development of periodontitis. These included *P. intermedia*, *T. forsythia*, *T. denticola*, and *P. gingivalis.* Our results suggest that systemic antibiotics prescribed for whatever purpose may also have an impact on the levels of these specific periodontal pathogens. We obtained similar results from both subgingival and saliva analyses, although two different methods were used, i.e., DNA-DNA hybridisation and qPCR. However, it remains unclear if this impact is sustained for more than 1 year after the use of antibiotics. In addition, we investigated the effect of the antibiotics only on the bacterial levels, although they may also affect the microbial activity, gene expression, protein synthesis, metabolite content, and translocation (Ferrer et al., 2017). The antibiotics had only a marginal effect on either saliva or serum antibody levels against the species mentioned above. The effects of the antibiotics on the bacterial levels were relatively small. Since no species were actually eradicated, seroconversion was not to be expected.

As expected, use of quinolones was strongly associated with subgingival gram-negative species as well as the ratio of Firmicutes/Bacteroidetes or gram-positives/gram-negatives. This was reflected also in saliva LPSactivity, which was inversely associated with the use of quinolones. Similarly, nonsignificant trend was seen also in serum LPS activity. We have shown earlier that the subgingival bacterial species predict saliva LPS activity and that saliva LPS correlates mildly with serum LPS activity, especially among periodontitis patients (Liljestrand et al., 2017). Thus, it is plausible that saliva LPS decreases when the number of its source species decline, and possibly also the trends seen in serum LPS activity follow the changes in gut microbiome after using antibiotics. However, the declining trend in both serum and saliva was short term and strongest only up to 1 month since the antibiotic course. LPS is effectively neutralised from the circulation by the liver and has a half-life between 2 and 4 min in mice (Yao et al., 2016). Unfortunately, our study design did not enable further investigations since the sampling was done only once. The gut is the main source of circulating LPS, but the oral microbiome may also contribute to its levels either directly to circulation or saliva or indirectly by affecting the gut microbiome composition (Olsen and Yamazaki, 2019; Schmidt et al., 2019). LPS is an important virulence factor and a putative mediator between oral and systemic health (Pussinen et al., 2021). LPS-activity is an independent risk factor for incident cardiometabolic diseases (Pussinen et al., 2021) and, interestingly, high LPS combined with a high number of antibiotic purchases has an additive effect on the risk of incident coronary heart disease (Simonsen et al., 2020).

We want to acknowledge some strengths and limitations of the study. The strengths include the well-characterised population and multifaceted laboratory results using plaque, saliva, and serum determinations arising both from bacteria and the host. The limitations include a *post-hoc* nature of the analyses accompanied by multiple testing, and the results need to be interpreted with caution. However, we employed several different approaches to solve the same research question resulting in similar results. For example, the levels of the same species both in saliva and subgingival plaque declined after using antibiotics despite totally different analysis methods. Our study data included only the use of systemic antimicrobials class J according to the ATC classification. Therefore, data on the use of metronidazole is missing, since it is classified as treating parasite infection (class P) in ATC classification system. Due to its adjunctive use to treat some periodontitis patients, there are ample of investigations on its effects on periodontal microbiota. Other limitations are the missing information on details of previous dental/periodontal treatments and the uncertainty, whether the patients really consumed the purchased antibiotic courses or what were the clinical implications. The main limitation is the targeted microbiome analyses using somewhat out-of-date methodology. For instance, the phyla were constructed by summing up levels of several species whose number differed. Firmicutes included the highest number of species (*n* = 32), while Treponemas included only two species. Nevertheless, the results are novel and with literature search we did not find any corresponding studies. Our study may be considered an opening for further studies using untargeted characterisation of the microbiome.

As a conclusion, the results show that systemic antibiotics influence the serology of periodontitis only marginally but have clear beneficial effects on periodontal inflammation and oral microbiota composition up to 5–8 months even without mechanical removal of biofilm. Thus, the effect may be shorter compared with results published in the meta-analysis (Teughels et al., 2020) for the adjunctive use of systemic antimicrobials with SRP. Using antibiotics as a monotherapy is not advisable, since SRP is highly effective in treatment of periodontitis and additional use of antibiotics increases resistance and may have adverse effects on the health of the patient.

## Data Availability Statement

The original contributions presented in the study are included in the article/**Supplementary Material**. Further inquiries can be directed to the corresponding author.

## Ethics Statement

The studies involving human participants were reviewed and approved by the Helsinki University Hospital Ethics Committee (Reg. no. 106/2007). The patients/participants provided their written informed consent to participate in this study.

## Author Contributions

EK, LL, MP, and AS were responsible of the data acquisition and writing the manuscript. PM and KB performed the clinical oral examinations. SH was responsible for the saliva antibody analyses and critical revision of the manuscript. RP was responsible for the subgingival microbiome analyses. JS is principal investigator of the Corogene cohort. SP and PP are principal investigators of the Parogene cohort. PP was responsible for the clinical laboratory and statistical analyses. All authors contributed to the article and approved the submitted version.

## Funding

P.J.P. received funding from the Finnish Dental Society Apollonia, the Yrjö Jahnsson foundation, the Sigrid Juselius foundation, the Aarne Koskelo foundation, the Foundation for Cardiovascular research, and the Academy of Finland (#1340750).

## Conflict of Interest

The authors declare that the research was conducted in the absence of any commercial or financial relationships that could be construed as a potential conflict of interest.

## Publisher’s Note

All claims expressed in this article are solely those of the authors and do not necessarily represent those of their affiliated organizations, or those of the publisher, the editors and the reviewers. Any product that may be evaluated in this article, or claim that may be made by its manufacturer, is not guaranteed or endorsed by the publisher.
