# Supplementary material for: Systemic Antibiotics Influence Periodontal Parameters and Oral Microbiota, But Not Serological Markers
Source: Front Cell Infect Microbiol. 2021 Dec 24;11:774665. doi: 10.3389/fcimb.2021.774665 (PMC8738095; doi:10.3389/fcimb.2021.774665)
Supplement: Supplementary file 1 [file DataSheet_1.pdf]

Supplementary table 1. Characteristics of different antibiotic classes

Antibiotic class (ATC-group)	Action mechanism	Spectrum
Tetracyclines (J01A)	Inhibitiom of protein synthesis	Broad
Penicillins (J01C)	Inhibition of cell wall synthesis	Narrow, Gram-positive
Cephalosporins (J01D)	Inhibition of cell wall synthesis	Intermediate, Gram-positive, Gram-negative
Trimethoprim (J01E)	Inhibition on DNA synthesis	Narrow, Gram-negative, Gram-positive
Macrolides / Lincomycin (J01F)	Inhibition of protein synthesis	Narrow, Gram-positive, Gram-negative
Quinolones (J01M)	Interference of DNA synthesis	Broad

ATC indicates Anatomical Therapeutic Chemical Classification

Supplementary table 2. List of the bacterial species studied with DNA-DNA checkerboard method

Gram positive-species	Phylum	Aerobe/anaerobe
Actinomyces israelii	Actinobacteria	*obligate anaerobe*
Actinomyces naeslundii	Actinobacteria	*facultatively anaerobic*
Actinomyces neuii	Actinobacteria	*facultatively anaerobic*
Actinomyces odontolyticus	Actinobacteria	*facultatively anaerobic*
Actinomyces oris sp. nov	Actinobacteria	*facultatively anaerobic*
Atopobium parvulum	Actinobacteria	*obligate anaerobe*
Atopobium vaginae	Actinobacteria	*obligate anaerobe*
Bifidobacterium biavatii	Actinobacteria	*anaerobic*
Bifidobacterium bifidum	Actinobacteria	*anaerobic*
Bifidobacterium breve	Actinobacteria	*anaerobic*
Bifidobacterium longum	Actinobacteria	*anaerobic*
Corynebacterium nigricans	Actinobacteria	*aerobic/facultatively anaerobic*
Corynebacterium pseudogenitalium	Actinobacteria	*aerobic/facultatively anaerobic*
Cutibacterium acnes (formerly Propionibacterium acnes)	Actinobacteria	*aerotolerant anaerobic*
*Gardnerella vaginalis**	Actinobacteria	*facultatively anaerobic*
Varibaculum cambriensis	Actinobacteria	*anaerobic*
Aerococcus christensenii	Firmicutes	*aerobic*
Anaerococcus vaginalis	Firmicutes	*obligate anaerobe*
Enterococcus faecalis	Firmicutes	*facultatively anaerobic*
Eubacterium saburreum	Firmicutes	*obligate anaerobe*
Lactobacillus acidophilus	Firmicutes	*microaerophilic/aerotolerant anaerobic*
Lactobacillus crispatus	Firmicutes	*microaerophilic/aerotolerant anaerobic*
Lactobacillus gasseri	Firmicutes	*microaerophilic/aerotolerant anaerobic*
Lactobacillus iners	Firmicutes	*microaerophilic/aerotolerant anaerobic*
Lactobacillus jensenii	Firmicutes	*microaerophilic/aerotolerant anaerobic*
Lactobacillus vaginalis	Firmicutes	*microaerophilic/aerotolerant anaerobic*
Parvimonas micra	Firmicutes	*anaerobic*
Peptoniphilus sp.	Firmicutes	*anaerobic*
Peptostreptococcus anaerobius	Firmicutes	*anaerobic*

Staphylococcus aureus	Firmicutes	*facultative anaerobe*
Staphylococcus aureus (gelbe Kultur)	Firmicutes	*facultative anaerobe*
Staphylococcus aureus (weisse Kultur)	Firmicutes	*facultative anaerobe*
Staphylococcus aureus subsp. anaerobius	Firmicutes	*facultative anaerobe*
Staphylococcus epidermis	Firmicutes	*facultative anaerobe*
Staphylococcus haemolyticus	Firmicutes	*facultative anaerobe*
Streptococcus agalactiae	Firmicutes	*facultative anaerobe*
Streptococcus anginosus	Firmicutes	*facultative anaerobe*
Streptococcus constellatus	Firmicutes	*facultative anaerobe*
Streptococcus gordonii	Firmicutes	*facultative anaerobe*
Streptococcus intermedius	Firmicutes	*facultative anaerobe*
Streptococcus mitis	Firmicutes	*facultative anaerobe*
Streptococcus mutans	Firmicutes	*facultative anaerobe*
Streptococcus oralis	Firmicutes	*facultative anaerobe*
Streptococcus pneumoniae	Firmicutes	*facultative anaerobe*
Streptococcus sanguis	Firmicutes	*facultative anaerobe*
**Gram-negative species**	**Phylum**	**Aerobe/anaerobe**
*Mobiluncus curtisii**	Actinobacteria	*anaerobic*
*Mobiluncus mulieris**	Actinobacteria	*anaerobic*
Bacteroides ureolyticus	Bacteroidetes	*anaerobic*
Capnocytophaga gingivalis	Bacteroidetes	*anaerobic*
Capnocytophaga ochracea	Bacteroidetes	*anaerobic*
Capnocytophaga sputigena	Bacteroidetes	*anaerobic*
Porphyromonas endodontalis	Bacteroidetes	*anaerobic*
Porphyromonas gingivalis	Bacteroidetes	*anaerobic*
Prevotella bivia	Bacteroidetes	*anaerobic*
Prevotella disiens	Bacteroidetes	*anaerobic*
Prevotella intermedia	Bacteroidetes	*anaerobic*
Prevotella melaninogenica	Bacteroidetes	*anaerobic*
Tannerella forsythia	Bacteroidetes	*anaerobic*
Dialister sp.	Firmicutes	*anaerobic*
Selenomonas noxia	Firmicutes	*anaerobic*

Veillonella parvula	Firmicutes	*anaerobic*
Fusobacterium nucleatum sp. nucleatum	Fusobacteria	*anaerobic*
Fusobacterium nucleatum sp. polymorphum	Fusobacteria	*anaerobic*
Fusobacterium nucleatum sp. fusiforme/Vincentii	Fusobacteria	*anaerobic*
Fusobacterium periodonticum	Fusobacteria	*anaerobic*
Leptotrichia buccalis	Fusobacteria	*anaerobic*
Aggrecatibacter actinomycetemcomitans	Proteobacteria	*facultative anaerobe*
Campylobacter gracilis	Proteobacteria	*microaerophilic ?*
Campylobacter rectus	Proteobacteria	*facultative anaerobe*
Campylobacter showae	Proteobacteria	*facultative anaerobe*
Eikenella corrodens	Proteobacteria	*facultative anaerobic*
Escherichia coli	Proteobacteria	*facultative anaerobic*
Haemophilus influenzae	Proteobacteria	*facultative anaerobe*
Helicobacter pylori	Proteobacteria	*microaerophilic*
Neisseria mucosa	Proteobacteria	*microaerophilic ?*
Proteus mirabilis	Proteobacteria	*facultatively anaerobic*
Pseudomonas aeruginosa	Proteobacteria	*anaerobic*
Treponema denticola	Spirochaetes	*anaerobic*
Treponema socranskii	Spirochaetes	*anaerobic*

* can also be defined as Gram variable

Supplemental Table 3. Characteristics of the population stratified by sex and the use of antibiotics during the preceding year.

**Parameter**		**No antibiotics (n=244)**			**Antibiotics (n=261)**		
		**Men (n=163)**	**Women (n=81)**	**p-value[1]**	**Men (n=165)**	**Women (n=96)**	**p-value[1]**
Age (years)		62.7 (9.2)	65.9 (8.9)	**0.012**	63.1 (8.2)	62.4 (10.3)	0.557
BMI (kg/m^2)		27.4 (4.2)	27.3 (5.4)	0.860	28.4 (4.9)	28.0 (6.1)	0.557
Number of teeth	x-ray	20.0 (9.3)	18.7 (9.0)	0.310	20.2 (8.7)	20.5 (8.3)	0.813
Caries (n of teeth)[2]	x-ray	1.10 (1.83)	0.87 (1.35)	0.349	0.97 (1.25)	0.85 (1.36)	0.476
Apical rarefactions (n of teeth)[2]	x-ray	0.49 (1.09)	0.43 (1.35)	0.600	0.29 (0.56)	0.28 (0.62)	0.899
BOP (% of sites)[2]	clinical	43.1 (18.9)	38.5 (20.2)	0.064	34.2 (17.1)	32.5 (18.4)	0.460
PPD≥4 mm (n of sites)[2]	clinical	15.5 (15.0)	11.0 (11.2)	0.060	13.5 (12.1)	9.9 (12.0)	**0.024**
PPD≥6 mm (n of sites)[2]	clinical	5.19 (11.3)	2.39 (5.42)	0.105	2.82 (6.32)	1.91 (6.47)	0.278
PIBI[2]	clinical	25.9 (32.3)	15.8 (20.7)	**0.011**	19.2 (20.9)	13.8 (21.8)	0.061
				**p-value[3]**			**p-value[3]**
Smoking (ever)[4]		95 (62.1)	23 (30.7)	**<0.001**	94 (61.0)	38 (42.7)	**0.006**
Periodontal treatment[4]		7 (4.8)	11 (14.7)	**0.011**	25 (17.0)	12 (14.1)	0.563
Diabetes		29 (17.8)	19 (23.5)	0.294	48 (29.6)	22 (23.2)	0.261
Alveolar bone loss	No	32 (20.8)	16 (21.6)	0.679	41 (26.3)	24 (26.7)	0.163
	Mild	62 (40.3)	35 (47.3)		67 (42.9)	49 (54.4)	
	Moderate	46 (29.9)	17 (23.0)		42 (26.9)	16 (17.8)	
	Severe	14 (9.1)	6 (8.1)		6 (3.8)	1 (1.1)	
Edentulous		10 (6.1)	7 (8.6)	0.469	8 (4.8)	7 (7.3)	0.413

[1] t-test; [2] log-transformation, mean and SD after back-transformation; [3] Chi-square; [4] Based on questionnaire on smoking and response to a question, whether the patient has ever received any periodontal treatment.

Supplementary table 4. Serum and saliva antibody levels in antibiotic-users and non-users during the preceding year.

Antigen	Antibody	Serum			Saliva		
		Non-users	Users		Non-users	Users	
		Mean (SD)		p[1]	Mean (SD)		p[1]
A. actinomyce-temcomitans	IgA	-0.12 (0.37)	-0.26 (0.36)	0.008	3.59 (0.34)	3.56 (0.31)	0.313
	IgG	-0.15 (0.31)	-0.22 (0.38)	0.026	4.05 (0.58)	3.91 (0.52)	0.007
P. gingivalis	IgA	-0.37 (0.54)	-0.41 (0.54)	0.388	3.80 (0.39)	3.74 (0.38)	0.168
	IgG	0.05 (0.33)	0.04 (0.32)	0.796	3.56 (0.51)	3.53 (0.49)	0.545
P. endodontalis	IgA	-0.29 (0.43)	-0.30 (0.37)	0.848	3.86 (0.28)	3.84 (0.30)	0.319
	IgG	0.11 (0.28)	0.09 (0.28)	0.426	3.46 (0.37)	3.46 (0.40)	0.878
P. intermedia	IgA	-0.21 (0.26)	-0.25 (0.30)	0.066	3.48 (0.32)	3.52 (0.33)	0.205
	IgG	-0.36 (0.25)	-0.36 (0.24)	0.976	3.25 (0.35)	3.26 (0.36)	0.630
T. forsythia	IgA	-0.71 (0.23)	-0.73 (0.35)	0.677	3.86 (0.25)	3.87 (0.26)	0.476
	IgG	-0.96 (0.34)	-0.96 (0.35)	0.915	3.39 (0.36)	3.37 (0.35)	0.743

[1] t-test; all antibody levels are log-transformed; the unit of serum antibody levels is EU (ELISA units) and saliva antibody levels RLU/100 ms (relative light units).

Supplementary table 5. Association of saliva and serum LPS-activity with subgingival phyla.

	LPS-activity	
	**Saliva**	**Serum**
	**B (SE), p-value**	
Actinobacteria	0.040 (0.072), 0.575	-0.029 (0.040), 0.476
Bacteroidetes	**0.142 (0.065), 0.030**	-0.021 (0.037), 0.574
Firmicutes	0.033 (0.094), 0.728	-0.078 (0.053), 0.138
Fusobacteria	0.099 (0.062), 0.110	-0.018 (0.035), 0.614
Proteobacteria	0.122 (0.070), 0.082	-0.020 (0.040), 0.611
Spirochaetes	**0.134 (0.054), 0.013**	-0.001 (0.030), 0.967
Firmicutes/Bacteroidetes	**-0.330 (0.108), 0.002**	-0.015 (0.061), 0.800
**Gram-positives**	**-0.464 (0.190), 0.015**	**-0.239 (0.107), 0.026**
**Gram-negatives**	**0.558 (0.179), 0.002**	0.167 (0.102), 0.102
**Gram-positives/gram-negatives**	**-0.534 (0.178), 0.003**	**-0.186 (0.101), 0.045**

Linear regression model adjusted for age and sex. All bacterial and LPS levels are log-transformed. Gram-positives comprise a sum of 45 and gram-negative a sum of 33 species.

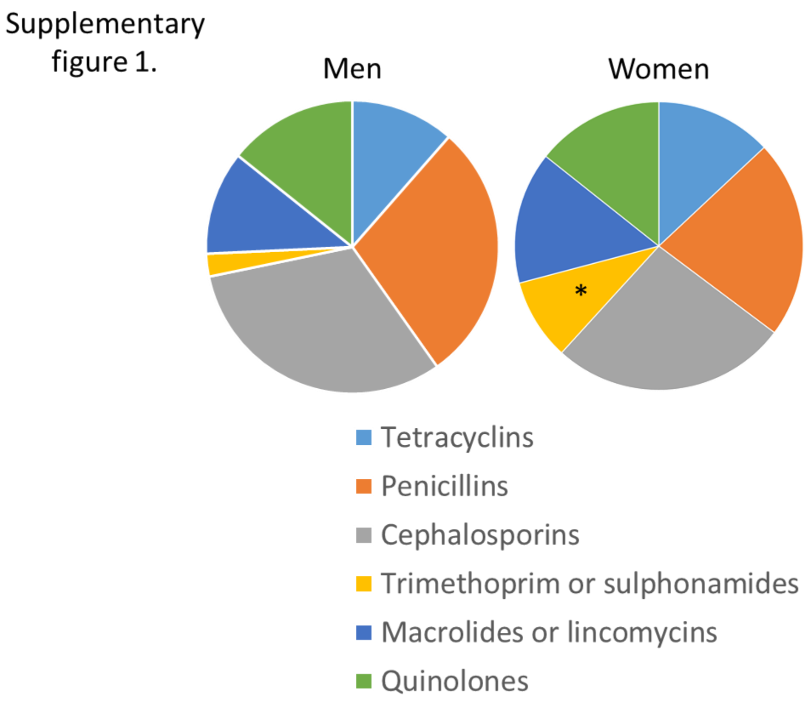

Supplementary figure 1. Antibiotic types in men and women. Two hundred sixty-one (51.7%) patients were prescribed with antibiotics during the preceding 1 year. Frequencies of antibiotic types are shown. P-values are analysed by t-test and the asterisk depicts $p<0.05$ between men and women.

Supplementary Figure 2

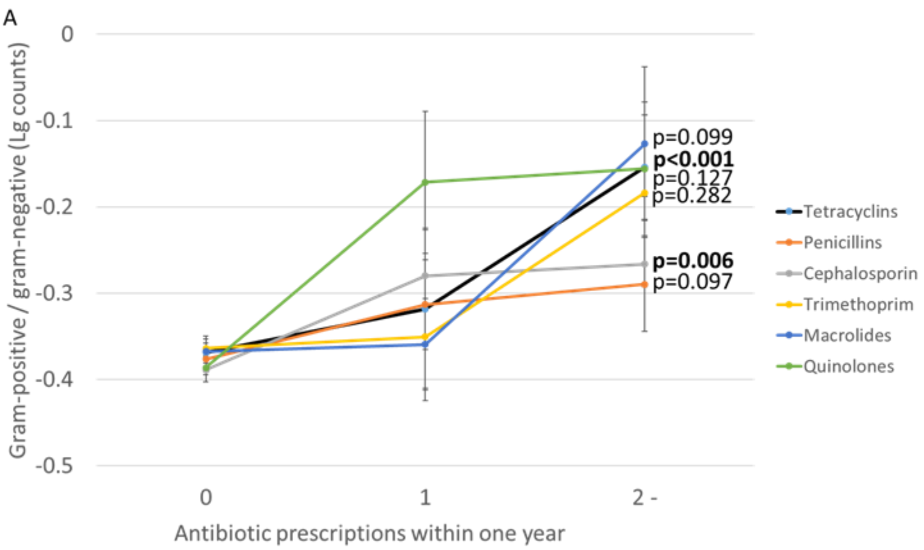

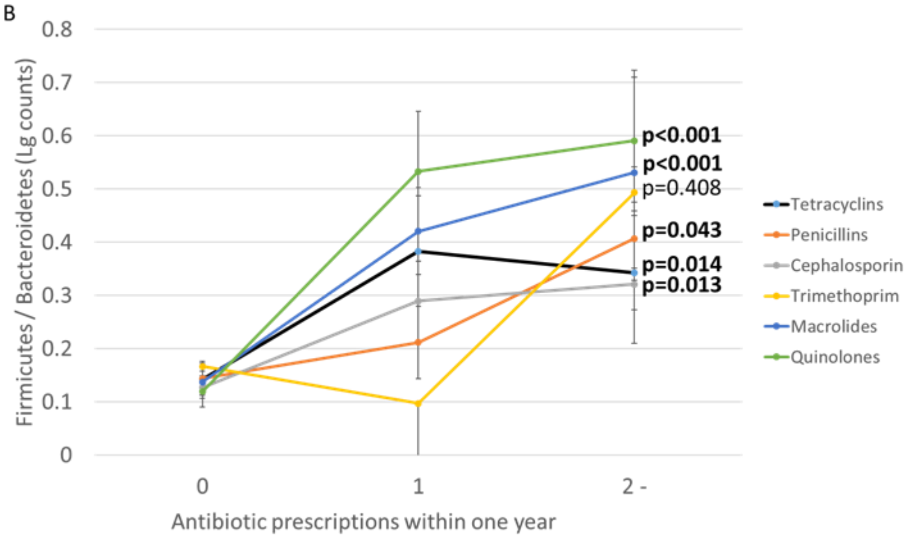

Supplementary figure 2. Effect of antibiotic courses on subgingival microbiota. Mean values of log-transformed bacterial counts on with the SE are shown. The bacteria are classified as ratios of: A) Gram-positive/gram-negative, and B) Firmicutes/Bacteroidetes. The p-values are weighted linear terms from ANOVA.
